# Brain structural changes in rural communities and disadvantaged neighborhoods: preliminary exploration in older adults from Southern Nevada

**DOI:** 10.1002/alz.089457

**Published:** 2025-01-09

**Authors:** Xiaowei Zhuang, Dietmar Cordes, Jessica ZK Caldwell, Andrew R. Bender, Justin B Miller

**Affiliations:** ^1^ Cleveland Clinic Lou Ruvo Center for Brain Health, Las Vegas, NV USA; ^2^ University of Nevada Las Vegas, Las Vegas, NV USA

## Abstract

**Background:**

Rural‐dwelling older adults are at increased risk for Alzheimer’s disease (AD) and related dementia. Identifying how rural living and neighborhood disadvantage affect neurobiology may help to understand rural‐urban disparities in AD and promote healthy aging in rural communities. In this study, we characterize rural‐urban differences in cortical thickness (CT), and the association of regional CT with neighborhood disadvantage, using both clinically normal and impaired older adults.

**Method:**

We assessed cross‐sectional data from 71 rural‐dwelling (61.97% women, 71.13±6.45 years old) and 87 urban‐dwelling (43.67% women, 72.16±6.88 years old) older adults from the Nevada Exploratory Alzheimer’s Disease Research Center and the Nevada Center for Neurodegeneration and Translational Neuroscience. The Rural‐Urban Commuting Area (RUCA) Codes score was used to determine rural‐urban residency status, and the Area Deprivation Index (ADI) was used to characterize neighborhood disadvantage. Clinical impairment status was determined by expert consensus review, and FreeSurfer‐derived CT measures were calculated from 3T structural magnetic resonance images (MRI). Analysis of covariance was used to test whether each CT measure was associated with rural‐urban living, clinical impairment status, or their interactions. Linear regressions were fitted to test the association between CT measures and ADI in clinically normal and impaired participants.

**Result:**

For temporal and inferior frontal regions, rural‐dwelling older adults demonstrated a thinner cortex than urban older adults, and these rural‐urban differences were more pronounced in clinically normal than impaired participants. Cortical thinning of temporal regions was further correlated with advanced neighborhood disadvantages. In contrast, for superior frontal, parietal and occipital regions, rural participants showed thicker cortex than urban participants and these differences were greater in clinically impaired participants.

**Conclusions:**

We observed extensive rural‐urban differences on CT measures with mixed directions, suggesting a complex interplay between community contexts and neurobiology in clinically normal and impaired older adults. More importantly, for regions involved in memory that are vulnerable to AD, our results suggest that living in rural areas and less advantaged neighborhoods might negatively affect subjects’ brain structures; but once clinical impairment onsets, impairment effect on these neurobiological measures tend be more dominant than the residency effect.

